# Association among Noncoding-RNAs, APRO Family Proteins, and Gut Microbiota in the Development of Breast Cancer

**DOI:** 10.32604/or.2025.062810

**Published:** 2025-08-28

**Authors:** Akari Fukumoto, Satoru Matsuda

**Affiliations:** Department of Food Science and Nutrition, Nara Women’s University, Kita-Uoya Nishimachi, Nara, 630-8506, Japan

**Keywords:** Breast cancer, cancer therapy, non-coding RNAs (ncRNAs), micro RNA, gut microbiota, anti-proliferative (APRO) family protein

## Abstract

The non-coding RNAs (ncRNAs) are a family of single-stranded RNAs that have become recognized as crucial gene expression regulators in normal and cancer cell biology. The gut microbiota, which consists of several different bacteria, can actively contribute to the regulation of host metabolism, immunity, and inflammation. Roles of ncRNAs and gut microbiota could significantly interact with each other to regulate the growth of various types of cancer. In particular, a causal relationship among ncRNAs, gut microbiota, and immune cells has been shown for their potential importance in the development of breast cancer. Alteration of ncRNA expression and/or gut microbiota profiles could also influence several intracellular signaling pathways with the function of anti-proliferative (APRO) family proteins associated with the malignancy. Targeting ncRNAs and/or APRO family proteins for the treatment of various cancers has been revealed with novel immune therapies. Here, the most recent studies to underline the key role of ncRNAs, APRO family proteins, and gut microbiota in breast cancer progression have been discussed. For more effective breast cancer therapy, it would be imperative to figure out the collective mechanism of ncRNAs, APRO family proteins, and gut microbiota.

## 1 Introduction

Breast cancer is the most prevalent cancer afflicting women globally. The incidence rate ascends with age, and more than 80% of breast cancer cases are identified in women above 50 years old worldwide [1]. Breast cancer may be a complicated and heterogeneous disorder, which exhibits the highest morbidity among female cancers [2,3]. Although there are distinct genetic risk factors such as tumor suppressor BRCA1/2 gene mutations and various environmental risk factors, there might be other unidentified risk factors for the majority of sporadic cases [4]. In recent years, the gut microbiota has been garnering significant attention from researchers [5]. An epidemiological study had demonstrated that the gut microbiota may increase to about 20% of malignant tumors [6]. In addition, the connection between the composition of the gut microbiota and the aggressiveness of cancers has been underlined [7]. These relationships have been reported during the function of *Helicobacter pylori* in gastric cancer and that of *Fusobacterium* in colon cancer for the promotion of carcinogenesis [8,9]. Interestingly, it has been shown that chronic bacterial infection in the bladder could change the bladder epithelial cells to cancerous cells [10]. Bladder bacterial infection may increase the risk of gene mutation and malignant transformation of cells [11].

Gut microbiota may crucially affect many aspects of individual biology from nutrient gaining to immunological function in the host. The gut microbiota is a community of trillions of microorganisms that utilize dietary components to produce several available metabolites that may influence the host’s health. Some metabolites could underlie the relationship between the gut and distal organs [12]. The gut microbiota also plays crucial roles in immune modulation and the maintenance of body homeostasis [13]. The composition of gut microbiota is closely related to the gut environment, which might include the redox state, pH, nutrients, and temperature of the host [14]. Bacterial infection can activate cancer-promoting signaling pathways including nuclear factor-kappa B (NF-κB) signaling, promoting the growth, survival, and metastasis of cancer cells [15]. The link between gut microbiota and breast cancer has firstly restricted from epidemiological studies [16]. Afterward, the interaction between the host and gut microbiota can form a complex and intricate regulatory network. Notably, non-coding RNAs (ncRNAs) have emerged as important mediators in this communication.

The role of ncRNAs in the interaction between host and gut microbiota as well as their influence on the host homeostasis and the host carcinogenesis is attracting increasing attention, suggesting a further interaction between ncRNAs and gut microbiota with carcinogenesis. By underlying host response mechanism to metabolic signals of gut microbial metabolites, therefore, several ncRNA expressions could be modulated. Interestingly, developmental patterns of mammary gland ncRNAs may provide clues to their dysregulated role in breast cancer [17]. ncRNAs are functional RNA molecules that are not translated into proteins, which may include transfer RNAs, ribosomal RNAs, circular RNAs (circRNAs), micro RNAs (miRNAs), and long non-coding (lncRNAs) based on their length and structure. Among them, miRNAs and lncRNAs are currently the most intensively studied [18]. Around 90% of the human genome is transcribed into ncRNA [19,20]. The discovery of these ncRNAs is regarded as an important breakthrough in life sciences [19,20]. Several ncRNAs play crucial roles in cellular development, physiological functions, and disease progression [21,22]. Therefore, ncRNAs have been rigorously explored for their roles in regulating human diseases including cancer development [23]. Now, ncRNAs have been providing them with encouraged candidates for the tool of cancer therapeutics [24]. Also, the gut microbiota can be altered for the prevention of various diseases including cancer by dietary interventions via the modification of ncRNAs. Here, we discuss the key role of ncRNAs, APRO family proteins, and gut microbiota mainly in the development of breast cancer, which would facilitate the study of pathogenesis for accelerating the process of therapeutic discovery against breast cancer.

## 2 Gut Microbiota and ncRNAs in the Development of Various Types of Cancer

**Figure 1:**
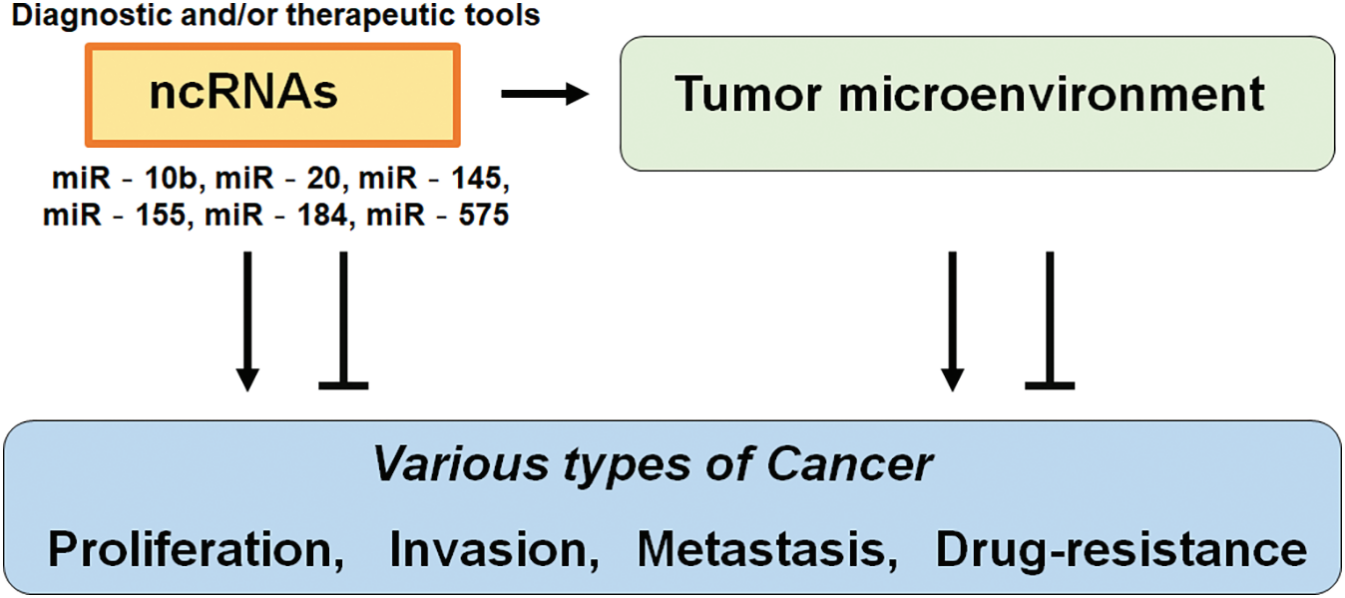
Illustration of the general action of non-coding RNAs (ncRNAs) to various types of cancer. Functions of ncRNAs have been proposed to relate to the proliferation, invasion, metastasis, and/or drug resistance of cancers. Consequently, certain ncRNAs could be diagnostic and/or therapeutic tools for cancers. The arrowhead means stimulation and/or augmentation, whereas the hammerhead represents inhibition.

Again, ncRNAs are RNA transcripts that are not translated, which may regulate biological processes such as cell proliferation, cell death, tumorigenesis, and immunity [25]. The miRNAs are short non-coding RNA sequences about 22 nucleotides long, which can bind to the 3′UTR of the specific mRNAs affecting the expression of mRNAs [26,27]. It has been suggested that serum miRNAs may serve as predictive prognostic biomarkers in various malignancies including breast cancer [28,29]. Numerous miRNAs including miR-10b, miR-20, miR-145, miR-155, and/or miR-575 have been identified as important in the progression of breast cancer [30]. In addition, several miRNAs have been found in the host serum [26–29] (Fig. 1) For instance, miR-184 may control the metastasis in triple-negative breast cancer by targeting the AKT/mammalian/mechanistic target of rapamycin (mTOR) pathway to cancer cell proliferation, invasion, and migration [31]. It has been suggested that abnormal expression levels of miRNAs could also influence the development of breast cancer [32]. In addition, increased expression of miR-25 may predict the better survival of breast cancer patients [33]. Moreover, some ncRNAs can be therapeutically targeted for targeting protein-coding mRNAs [34]. Therefore, understanding the specific signature of ncRNAs could help to understand the carcinogenic mechanisms of breast cancer for the development of the diagnosis and/or treatment. One of the challenges in the cancer therapy field is to clear the action of various ncRNAs, which is critical for developing its potential use as a biomarker and/or medical treatment target [23,35]. Fortunately, it has been demonstrated that some ncRNAs including the miR-25-3p can be used as a prognosis biomarker and a cell proliferation regulator in various types of cancer such as ovarian cancer, hepatoma, and esophageal squamous cell carcinoma [36–39].

Gut microbiota and ncRNAs can interact in the gut epithelial cells, leading to the activation of key signaling pathways and modulation of gene expression for the host cells [40]. The gut epithelial cells could secrete various types of major ncRNAs in the gut [41]. Host ncRNAs can be incorporated into several bacteria and specifically modify the bacterial transcripts, which possibly alleviate the symptoms of host colitis [41]. At the same time, the gut microbiota can control the expression of ncRNAs of gut epithelial cells, thereby modifying the homeostasis of gut microbiota [41]. The probiotics could therefore improve the damage to the gut by regulating the ncRNA expression [42]. For example, the *Lactobacillus fermentum* can increase the expression of miR-159 and/or miR-143, thereby maintaining the function of the intestinal barrier via their anti-inflammatory effects [42]. In addition, *A. muciniphila* can also increase the expression of miR-143 and/or miR-145, which may promote the gut barrier integrity [43]. After the inoculation with *S. enterica* to the gut, the expression of miR-214 may reduce, whereas that of miR-331-3p may increase, which can induce several immune responses against *S. enterica* [44]. Other pathogenic bacteria including *H. pylori* can also affect the expression of ncRNAs in the gut [45,46]. Interestingly, commensal bacteria can downregulate the expression of miR-10a in dendritic cells [47]. Gut microbiota and commensal bacteria might alter the mucosal immune response by regulating the mucosal innate response [47]. Therefore, the gut microbiota has a significant impact on the host immune homeostasis. Alterations in the composition of gut microbiota may also be associated with metabolic disorders including obesity, type 2 diabetes, and/or cardiovascular disease [48,49]. The gut microbiota homeostasis can be affected by inflammatory stressors such as lipopolysaccharides (LPS) and/or dietary ingredients [50]. An imbalanced population of gut microbiota may contribute to the compromised gut barrier integrity [51], which may allow bacteria to start systemic immune responses [52]. In addition, some bacteria such as *F. prausnitzii* can reduce the proliferation of colorectal cancer cells by producing butyrate. Then, the butyrate can inhibit *miR-92a* transcription, thus increasing the tumor suppressor p57 level to suppress the cancer activity [53]. The *P. bivia* may also be involved in the fermentation of dietary fibers to synthesize butyrate, serving a key role in intestinal homeostasis [54]. The butyrate could play a significant role in the development of human cancers via epigenetic action by inhibiting histone deacetylase 3 [55]. Therefore, fecal microbiota transplantation (FMT) is a possible strategy to modulate the gut microbiota for the production of butyrate to regulate the cancer-immune system [56]. In addition, the FMT could also alternate the gut microbiota for the production of some ncRNAs that can directly alter the expression of cancer-related genes to interfere with the development of several malignant tumors [57,58]. In line with this, dysbiosis can eventually lead to the dysregulation of gene expression in the mammary glands for the increased risk of breast cancer development [59]. These findings suggest that certain gut microbiota could alter the expression of cancer-related genes for the inhibition of various types of cancer.

## 3 APRO Family Proteins Involved in Breast Cancer

**Figure 2:**
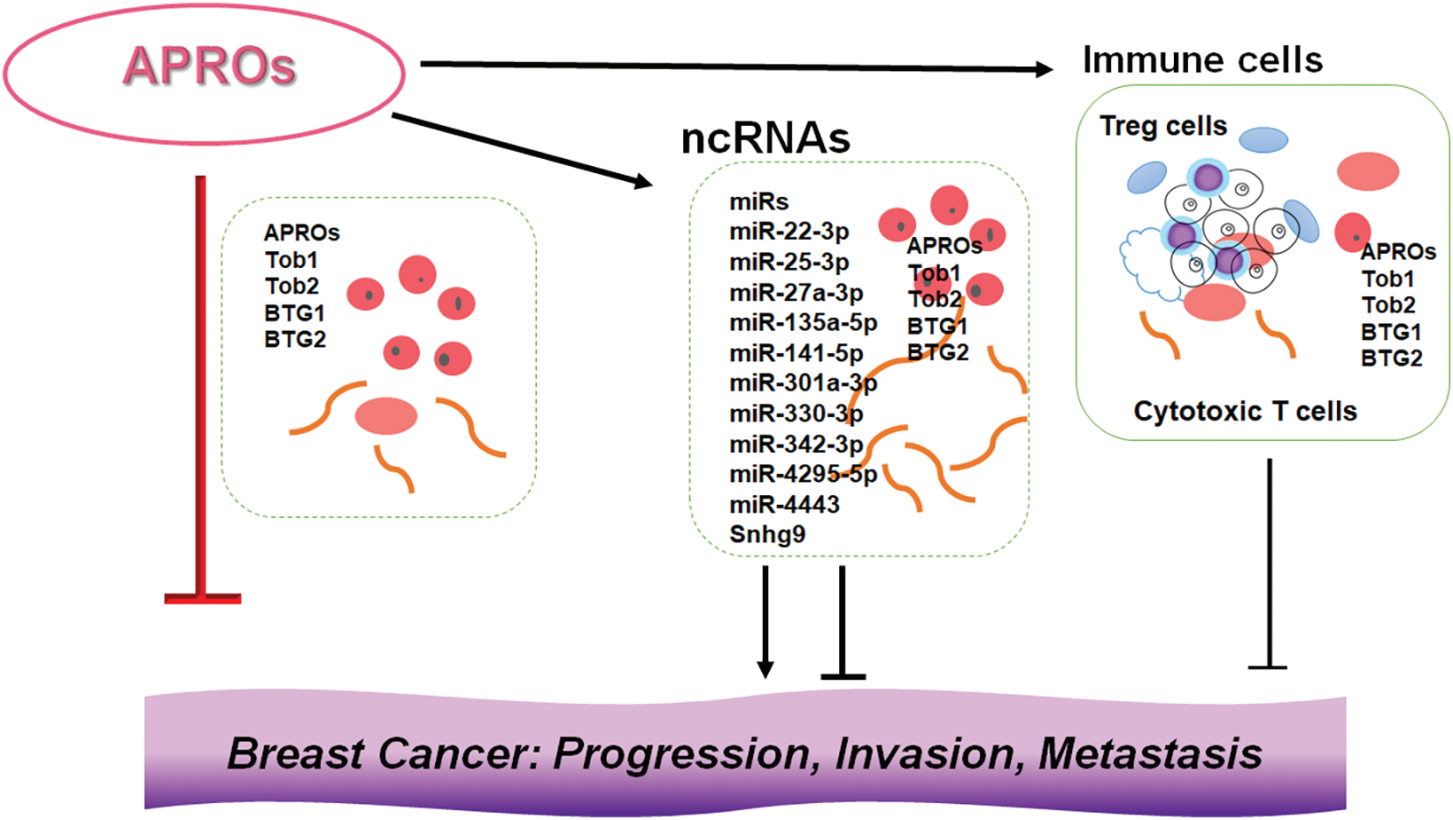
Illustration of the relationship among APRO family proteins, ncRNAs, and immune cells in the breast cancer development. Although APRO family proteins (APROs) could individually inhibit the progression, invasion, and/or metastasis of cancer cells, the other conditions such as expression levels of ncRNAs and/or the function of immune cells in the microenvironment of tumors may also contribute to the development of breast cancer. Indicated proteins and/or RNAs are examples. Arrowhead means stimulation, whereas hammerhead represents inhibition. Note that some critical pathways have been omitted for clarity. Abbreviation: APROs, APRO family proteins; miRs: microRNAs.

Breast cancer is a common malignancy in women worldwide [60]. The incidence of breast cancer has increased over the past few decades [61]. Although multidisciplinary therapies have been used for the treatment of breast cancer, the morbidity and mortality rates remain high and the prognosis of breast cancer remains poor [62]. Early diagnosis may result in a significant improvement in the disease prognosis and quality of life (QOL) of patients [63]. Previous findings have indicated that *the transducer of ERBB2, 1 (Tob1)* gene expression is downregulated in various cancers including breast cancer [64]. In addition, Tob1, which is ubiquitously expressed in human adult tissues, could work as a tumor suppressor in certain types of cancers [65]. The *Tob1* gene is located on chromosome 17q21 and encodes for a 45 kDa Tob1 protein [66]. Tob1 is a member of the APRO protein family. Tob1 is associated with the regulation of tumor cell proliferation and invasion, which may also contribute to the inhibition of cancer migration and metastasis [67]. It has been shown that *Tob1* knockdown can promote tumor cell migration and invasion in gastric and lung cancer cells [68,69]. In breast cancer cells, *Tob1* overexpression could induce the apoptosis of cancer cells [70]. In general, the *Tob1* is downregulated in breast cancer cells compared with normal cells, and the miR-25-3p knockdown can repress the proliferation of breast cancer cells by upregulating *Tob1* expression [70,71]. It has been shown that the *Tob1* gene may be a key determinant of survival in estrogen-dependent estrogen receptor-positive breast cells [71]. A previous study demonstrated that miR-25-3p functions as an oncogene and promotes proliferation via the induction of B-cell translocation gene 2 (BTG2), another member of the APRO protein family, in breast cancer [72]. Analysis of ncRNAs shows that the miR-25-3p is overexpressed in the serum of patients with breast cancer [73]. The B-cell translocation gene 1 (BTG1) could reverse the miR-22 for the inhibition of autophagy, while the miR-4295 could significantly promote the proliferation and migration of cancer cells via directing the BTG1 [74,75]. By inhibiting BTG1, miR-511 can strengthen the proliferation of human hepatoma cells, while miR-301 can promote the development of colitis-associated cancer [76,77]. In addition, the BTG1 may function as a direct target of miR-330-3p in hepatocellular carcinoma, which could reduce the cell viability, migration, and/or invasion, whereas promoting cancer cell apoptosis [78]. On the contrary, the miR-141-5p can enhance the proliferation and inhibit apoptosis in cervical cancer cells by targeting the *BTG1* gene [79]. In addition, the miR-301a-3p can promote the proliferation and invasion of nasopharyngeal carcinoma squamous cells also by targeting the *BTG1* gene [80]. In these ways, APRO family proteins including Tob1, BTG2, and BTG1 have been reported to control/regulate/promote cell proliferation, cell cycle progression, and/or cell differentiation in various types of cancer [81] (Fig. 2).

## 4 Roles of APRO Family Proteins in the Regulation of Cancer and Immune Cells

The APRO family may be associated with the initiation and progression of cancers. The APRO family includes 6 members: Tob1, Tob2, BTG1, BTG2, Abundant in Neuroepithelium Area (ANA), and BTG4, which some members are of significance for the prognosis of cancers [82–85]. Consequently, the expression of APRO family members is linked with patient prognosis. In addition, APRO family genes show significant association with immune infiltration, cancer cell stemness, and tumor microenvironment [82,84]. Several lines of evidence suggest that the quiescence of naïve T cells is tightly regulated by the activity of nuclear factors including Tob1 [86]. The Tob1 is also a transcriptional repressor, which has been shown to play a crucial role in keeping naïve T cells from excess proliferating [87]. Remarkably, the decreased *Tob1* expression at the mRNA and protein levels in immune cells has been confirmed in experimental autoimmune encephalomyelitis and/or multiple sclerosis [88]. Interestingly, it has been also reported that Tob1 participates in tumor occurrence as well as T-cell activation [89,90]. In addition, high expression of Tob1 could inhibit the proliferation of malignant pancreatic cells [91]. Therefore, some researchers suppose that Tob1 can be used as an independent indicator to evaluate the prognosis of patients with various types of cancer [92]. In the study of esophageal squamous cell carcinoma, down-regulated Tob1 expression may be correlated with the unfavorable prognosis of the patients [93]. Additionally, Tob1 has been uncovered to trigger autophagy to suppress cancer progression by activating the AKT/mTOR pathway [94]. BTG1 mutations may also disrupt a critical immune porter mechanism that can limit the B cell suitability during the antibody maturation in diffuse B cell lymphoma [95,96]. The overexpressed BTG2 could reduce the proliferation and migration of various types of cancer cells, which may also act as an effective target for the treatment of breast cancer [97,98]. Both BTG1 and BTG2 are closely correlated with the prognosis of cancer patients [99]. Consistently, the ANA overexpression could also inhibit the proliferation and invasion of various types of cancers including epithelial ovarian cancer [100].

Follicular B cells can induce the production of regulatory T (Treg) cells, further enhancing the immunosuppressive microenvironment. Tumor cells may favor the immunosuppressive microenvironment to evade immune surveillance, thereby aggravating the development of the cancer disease [101]. In general, during the immune response, the activation of T cells may be a crucial step that qualifies them to respond to various alien antigens. However, to keep the balance of the immune system for avoid excessive immune responses, some T cells are required to be in a dormant state, known as quiescent T cells [102]. Under specific conditions, activated T cells may also be suppressed to avoid autoimmune reactions. Tob1 could play a significant role in the development of these quiescent T cells as a negative regulator for T cell activation [103]. In addition, other APRO family proteins could also play a role in adaptive immunity by inhibiting immune cell proliferation/differentiation, thereby keeping immune cells in a quiescent state [87,104]. Therefore, it would be possible that several ncRNAs could activate immune cells against cancers via the inhibition of APRO family proteins (Fig. 2).

## 5 Gut Microbiota Could Modify the Development of Breast Cancer via the Alteration of ncRNAs and APRO Family Proteins

**Figure 3:**
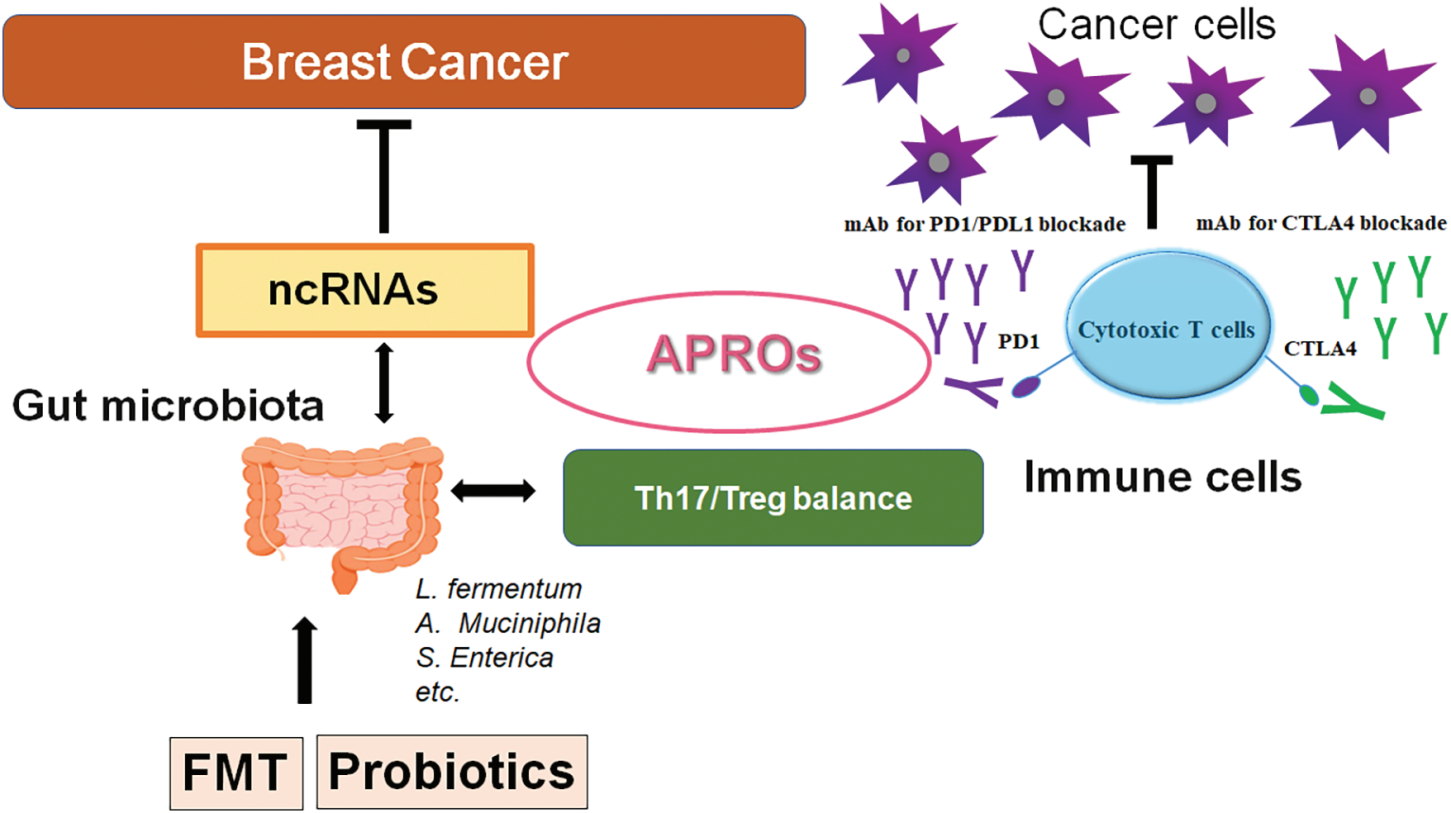
A schematic representation and hypothetical overview of the gut microbiota and ncRNAs therapy against breast cancers. Certain gut microbiota could contribute to the potentiation of the immune checkpoint immunotherapy with the improvement of Th17/Treg immune cell balance. Some kinds of probiotics and/or FMT could contribute to the alteration of the gut microbial community for playing valuable roles in cancer therapy with the alteration of ncRNAs and APRO family proteins. APRO family proteins could further stimulate the cytotoxic T cells. Examples of certain microbial species with several effects on immune responses have been shown. The arrowhead indicates stimulation (or bidirectional stimulation), whereas the hammerhead shows inhibition. Note that several important activities such as cytokine induction have been omitted for clarity. Abbreviation: APROs, APRO family proteins; miRNAs: microRNAs; PD1, programmed cell death protein 1; CTLA4, cytotoxic T-lymphocyte-associated protein 4.

The gut microbiome has been demonstrated to be associated with various types of cancer [105,106]. In particular, previous studies have provided evidence suggesting that immune cells possess fundamental links with both the gut microbiota and breast cancer [107,108]. Remarkably, gut microbiota could modulate the response to anti-PD-1 immunotherapy in breast cancer and melanoma patients [107,108]. Studies have demonstrated that dysbiosis could facilitate the development of malignant tumors by stimulating unregulated inflammatory responses, which may also suggest to play a crucial role in cancer prevention [109,110]. Given the function of gut microbiota in the process of inflammation-mediated carcinogenesis, it is predictable that specific gut microbiota or the relevant dieting might be correlated with the development of specific cancers [111,112]. Interestingly, flaxseed oil is among the highest plant-based sources of alpha-linolenic acid, which might reduce mammary tumor growth and tumor proliferation via the alteration of gut microbiota composition [113]. The flaxseed oil can also increase the serum levels of eicosapentaenoic acid and/or docosahexaenoic acid, which may improve the inflammation intensity [113]. For the occurrence and the development of breast cancer, the mechanism of the interaction between gut microbiota and immune cells might be complex, and the gut microbiota could affect carcinogenesis through immune cells in a variety of ways including inflammatory signaling with intestinal epithelium [114,115]. A causal relationship between gut microbiota, immune cells, and breast cancer, underlining the critical role of the gut microbiota in regulating immune responses and its potential importance in breast cancer has also been shown [116]. The gut microbiota is capable of generating several metabolites such as short-chain fatty acids (SCFAs), and lactic acid. These metabolites can regulate the immune response by activating immune cells, thus maintaining the immune homeostasis of the gut mucosa [117]. The SCFAs have been suggested to regulate the development of breast cancer. The microbiota could also reprogram the gut lipid metabolism by inhibiting the expression of lncRNA such as *Snhg9* in gut epithelial cells, which could promote cancer cell proliferation, migration, and invasion [118]. Patterns of mammary gland miRNA expression may also provide some clues to their dysregulated role in the development of breast cancer [17,119] (Fig. 2). Inflammation mediators can alter the gene and ncRNA expression in mammary cells, leading to the development of a cancer stem cell (CSC) phenotype, which can contribute to the onset of breast cancer [120]. By epigenetic regulation, the gut microbiota could also modify the host physiological process via the alteration of ncRNAs [121]. An intricate regulatory network exists between the host and the microbiota, and elucidating this complicated network may be required for future cancer research. Interactions of various ncRNAs with the gut microbiota would influence tumor development and therapy, as the gut microbiota with ncRNAs has been identified to possess a close association with the immune system [122]. For example, colonization of the gut microbiota can assist the neonatal immune system in establishing immune tolerance [122]. Gut microbiota could also influence the development of several immune organs such as the spleen and thymus [123], which can enhance the intrinsic immune defense system by stimulating Toll-like receptors and/or the complement system [124]. Again, gut microbiota could control adaptive immune responses by affecting the function of dendritic cells (DCs) as well as adjusting the antibody production of immune B cells [125]. These effects may be essential for the regulation of the host’s inflammatory responses. Interestingly, it has been shown that Tob1 can exhibit close associations with infiltrating Treg cells in pancreatic cancer, suggesting its involvement in the control of Treg cell function [126], which may reduce the number of Treg cells [127]. Treg cells can construct an immunosuppressive microenvironment through the secretion of immunosuppressive cytokines, thereby facilitating the escape of cancer cells from immune systemic cancer surveillance shown in urologic malignancies [128]. Although these findings have been obtained from non-breast cancers, we believe that further elucidation of the interaction between the host microbiota and ncRNAs involved in the regulation of immune surveillance might also clarify the phenotypic roles of breast cancer (Fig. 3).

## 6 Future Perspectives and Concluding Remarks

Traditional treatments for breast cancer mainly include radiotherapy, chemotherapy, surgical excision, and/or novel immunotherapies. Among the available treatments for breast cancer, the combination of cancer therapies is the first-line treatment [129]. High-risk patients of breast cancer may take simultaneous chemotherapy, which may include the PD-1/PD-L1 inhibitors [130]. Although these cancer therapies may play an imperative role in the treatment of breast cancers, which has several limitations with adverse effects. In contrast, the ncRNAs-related therapy has potential advantages over the conventional treatment. For example, ncRNA therapies may be specific reducing the impact on normal/noncancerous cells, which can interfere with the biological behavior of cancer cells from a specific perspective (Fig. 1). Therefore, ncRNA therapies may have superior safety with fewer adverse effects [131]. Interestingly, it has been found that many ncRNAs involved in metformin anticancer therapy have been identified [132]. The interaction between the host and the gut microbiota forms a complex and intricate regulatory network. Importantly, several ncRNAs have emerged as important mediators and/or outputs of this communication. The role of ncRNAs in host-microbiome interactions as well as their influence on breast cancers has been increasing attention. Clarifying these relationships with APRO family proteins would provide valuable insights into the prevention/treatment of breast cancers (Fig. 3).

## Data Availability

Data sharing not applicable to this article as no datasets were generated or analyzed during the current study.

## Abbreviations

ANA: Abundant in Neuroepithelium Area
BTG1: B-cell translocation gene 1
BTG2: B-cell translocation gene 2
circRNAs: Circular RNAs
CSC: Cancer stem cell
CTLA4: Cytotoxic T-lymphocyte-associated protein 4
DCs: Dendritic cells
FMT: Fecal microbiota transplantation
lncRNAs: Long non-coding
LPS: Lipopolysaccharide
miRNAs: Micro RNAs
mTOR: Mammalian/mechanistic target of rapamycin
ncRNAs: Non-coding RNAs
NF-κB: Nuclear factor-kappa B
PD1: Programmed cell death protein 1
QOL: Quality of life
ROS: Reactive oxygen species
SCFAs: Short-chain fatty acids
TGF-β: Transforming growth factor-β
Th17: T helper 17 cell
Tob1: Transducer of ERBB2, 1
Tob2: Transducer of ERBB2, 2
Treg: Regulatory T cell
